# Aging-related genes are potential prognostic biomarkers for patients with gliomas

**DOI:** 10.18632/aging.203008

**Published:** 2021-05-04

**Authors:** Gelei Xiao, Xiangyang Zhang, Xun Zhang, Yuanbing Chen, Zhiwei Xia, Hui Cao, Jun Huang, Quan Cheng

**Affiliations:** 1 Department of Neurosurgery, Xiangya Hospital, Central South University, Hunan, China; 2 Department of Oncology, Xiangya Hospital, Central South University, Hunan, China; 3 Department of Neurology, Hunan Aerospace Hospital, Changsha, Hunan, China; 4 Department of Psychiatry, The Second People’s Hospital of Hunan Province, Hunan, China; 5 The Hospital of Hunan University of Chinese Medicine, Hunan, China; 6 National Clinical Research Center for Geriatric Disorders, Xiangya Hospital, Central South University, Hunan, China

**Keywords:** aging, gliomas, aging-related genes, risk model

## Abstract

Aging has a significant role in the proliferation and development of cancers. This study explored the expression profiles, prognostic value, and potential roles of aging-related genes in gliomas. We designed risk score and cluster models based on aging-related genes and glioma cases using LASSO Cox regression analysis, consensus clustering analysis and univariate cox regression analyses. High risk score was related to malignant clinical features and poor prognosis based on 10 datasets, 2953 cases altogether. Genetic alterations analysis revealed that high risk scores were associated with genomic aberrations of aging-related oncogenes. GSVA analysis exhibited the potential function of the aging-related genes. More immune cell infiltration was found in high-risk group cases, and glioma patients in high-risk group may be more responsive to immunotherapy. Knock-down of CTSC, an aging-related gene, can inhibit cell cycle progression, colony formation, cell proliferation and increase cell senescence in glioma cell lines *in vitro*. Indeed, high expression of CTSC was associated with poor prognosis in glioma cases. In conclusion, this study revealed that aging-related genes have prognostic potential for glioma patients and further identified potential mechanisms for aging-related genes in tumorigenesis and progression in gliomas.

## INTRODUCTION

Glioma is the most common primary intracranial tumor, with an annual incidence of 6.6 per 100,000 individuals in the USA [1–3]. Adult gliomas can be subdivided into grades II–IV according to the WHO (World Health Organization) grading system based on their degree of malignancy [1–3]. Glioblastoma (GBM) is a malignant brain tumor and is the most frequent and aggressive form of glioma [1]. The treatment options for GBM (glioblastoma) patients include surgical resection, radiotherapy and chemotherapy [2, 4, 5]. Unfortunately, population-based studies have shown that the median patients survival times for these patients is only approximately 15 months [1, 4], and the five-year survival rates for glioblastoma is 6.8% [1, 6].

**Figure 1:**
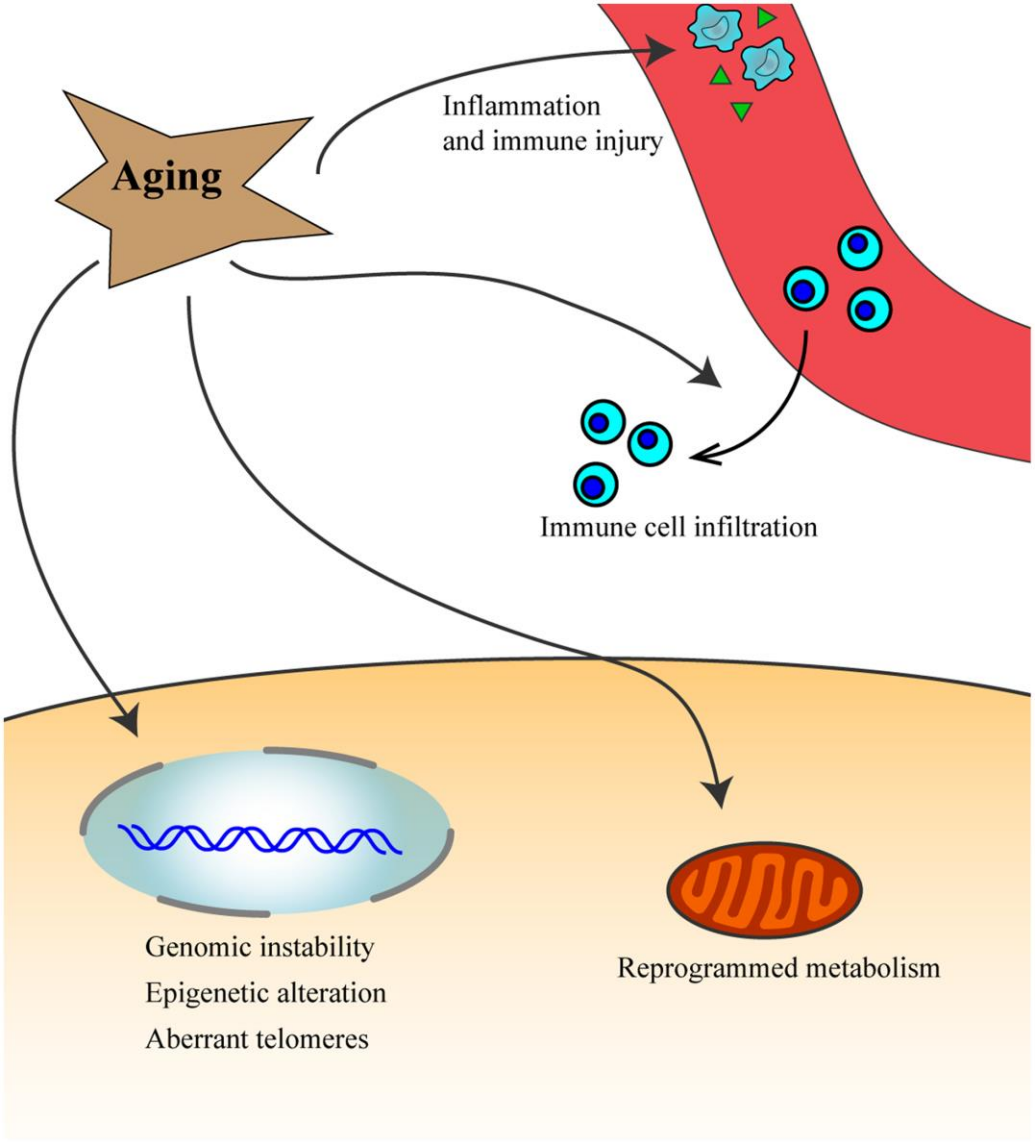
Aging process leads to inflammation and immune injury, increases genomic instability, epigenetic alteration, aberrant telomeres and affects immune cells infiltration, and reprogram metabolism, which might then promote the occurrence and development of cancer.

Aging is characterized by progressive and irreversible reduction in body functional integrity and homeostasis and is associated with increased vulnerability to death [7, 8]. Aging is associated with increased risk for several diseases such as cancer (Figure 1), neurodegenerative disease, and stroke [9, 10]. Cancer and aging share some hallmarks such as epigenetic alteration, reprogrammed metabolism, immune and inflammation injury and aberrant telomeres [10–13]. Furthermore, there are signaling pathways that are common between aging and cancer, such as the Arf/p53 pathway, AIM2-like receptors, and toll-like receptors [10, 14, 15]. Aging can also increase the risk for cancer through factors associated with immunity and inflammation [16, 17]. A lot of aging-related genes (eg: ERBB2, PTEN and P53) play important roles in cancer [18–20]. Cell senescence is closely associated with aging, with the accumulation of senescent cells in tissues triggering the aging process which affects the regenerative potential of stem cells. However, the senescence response is widely recognized as a potent barrier to the initiation of tumorigenesis and development of cancer [21–23].

The relationship among aging, senescence and cancer is not well known and there is need for further research to determine the association among the three factors [24, 25]. Aging-related genes such as CTSC and ARNTL are associated with several cancers. Previous studies have reported the role of CTSC in some cancers, but its relationship with aging and gliomas is not clear [26–28].

It is unclear if aging-related genes have a significant effect on gliomas and the mechanism underlying this effect. In this study, using data obtained from the CGGA (the Chinese Glioma Genome Atlas) and TCGA (the Cancer Genome Atlas), we determined if aging-related genes have any relationship with the molecular features, clinical features and prognosis of glioma patients. Results from this study may provide insight into potential therapeutic and diagnostic targets for gliomas.

## MATERIALS AND METHODS

### Data collection

The clinical information (age, gender, mutational status, isocitrate dehydrogenase (IDH), 1p/19q codelet, giloma grade, type and survival information) and mRNA expression profiles derived from TCGA (The Cancer Genome Atlas) (http://cancergenome.nih.gov) and the CGGA (Chinese Glioma Genome Atlas) (http://www.cgga.org.cn). The data obtained from TCGA was used as the training set, while the data obtained from CGGA was used as the validation set. Other glioma datasets were got from GEO (https://www.ncbi.nlm.nih.gov/geo/). Cases from IMvigor datasets were used to analysed the response to immunotherapy of cases with different risk score. The differential expression of aging-related genes was displayed using heat maps.

### Select aging-related genes and univariate cox regression analyses

We selected 321 aging-related genes by compiling 7 gene sets associated with aging from GSEA (https://www.gsea-msigdb.org). Of the 321 genes, we found 312 genes in the TCGA dataset. We then performed univariate cox regression analyses and identified 249 potential genes with close association with aging.

### Least absolute shrinkage and selection operator cox regression

Survival risk assessment model was construct using aging-related genes, by Least absolute shrinkage and selection operator (LASSO) Cox regression (lambda 1se) analysis. In light of the highest lambda value [29], the LASSO coefficients were calculated, using prognostic aging-related genes. Then the risk score model was constructed on the basis of LASSO coefficients. Relationship among clinical features, risk score and aging-related genes was displayed using box plots and heat maps.

### Consensus clustering analysis

Using consensus clustering analysis, the glioma cases in TCGA and CGGA were grouped into several subgroups according the mRNA expression levels of aging-related genes.

### Gene set variation analysis (GSVA)

Using “GSVA package”, functional enrichment analysis was performed as previously described [30] to reveal the mechanisms of action of the aging-related genes in the initiation and progression of gliomas.

### Analysis of genetic alterations

Analysis of somatic copy number alternations (SCNAs) and somatic mutations were conducted to investigate genetic alterations in gliomas based on TCGA database. These samples were divided into low and high-risk score groups based on the values of the risk scores. The 20 genes with the most mutations in the two groups were screened and displayed. Additionally, GISTIC 2.0 was used to compare SCNAs between the two groups, and gene copy number variation data downloaded from TCGA [31]. We also obtained the threshold copy number using GISTIC analysis.

### Survival analysis

Using Kaplan-Meier method and log-rank test, Survival curves were generated and compared. The OS (Overall Survival), PFI (Progression Free Survival), and DSS (Disease Free Survival) rates of cases in the high and low-risk groups were compared. We also conducted and compared survival analyses between the two clusters.

### Receiver operating characteristic (ROC)

The prediction value of clusters, risk score, grade and age was compared using ROC in some respects, including 5-year OS, 5-year PFI, 5-year DSS, IDH status, MGMT status, 1p19q codel status, subtypes.

### Cell culture and treatment

U251 cells and SHG-44 cells were cultured in DMEM medium (Sigma, USA, #D5796) supplemented with 10% Gibico FBS and 1% double antibody (Gibco, USA, #10099141). The media was changed every 2 - 3 days. When the cell confluence reached about 80%, trypsin (Beyotime Biotechnology, Shanghai, China, #C0201) was used to digest the cells and divide them into two groups for cell passage. The two types of logarithmic growth cells were grouped as follows: Control group: U251 cells and SHG-44 cells were not treated, siRNA-NC group: U251 cells and SHG-44 cells were transfected with siRNA-NC, siRNA-837 group: U251 cells and SHG-44 cells transfected siRNA-837 (Honor Gene, Changsha, China), siRNA-963 group: U251 cells and SHG-44 cells transfected siRNA-963 (Honor Gene, Changsha, China).

Serum-free DMEM (95 μL) was added into 8 sterile centrifuge tubes. Thereafter, 5 μL siRNa-NC and 5 μL Lip2000 were added into each centrifuge tube followed by the addition of siRNA-623, siRNA-837, and siRNA-963 into the corresponding centrifuge tube. The mixture was gently mixed and kept at room temperature for 5 min. Afterwards, the solutions in two tubes of same group were mixed and kept at room temperature for 20 min. Finally, the mixture was slowly added to the transfection hole and mixed. The cells were then incubated at 37° C, and the culture medium was changed after 6 h. Samples were collected for subsequent experiments after 48 h.

### Quantitative real time PCR

The mRNA was extracted using the RNeasy kit (Thermo, USA, #15596026) according to the manufacturer’s instruction. This was followed by the reverse-transcription of mRNA to cDNA using the mRNA reverse transcription kit (cwbio, Beijing, China, #CW2569). The primers sequences were generated using NCBI (table). The primers for H-actin were (5’-ACCCTGAAGTACCCCATCGAG-3’ (Forward), 5’-AGCACAGCCTGGATAGCAAC-3’ (Reverse)), While the primers for H-CTSC were (5’-GCTACTGACTTTCTTGCCTAAACCA-3’ (Forward), 5’-CAACAGAGCAGGAAACAAGACC-3’ (Reverse)). The thermocycler was set at 95° C for 10 min, followed by 40 cycles of 95° C for 15 s, and 60° C for 30 s. qRT-PCR was conducted using a Fluorescence quantitative RCP instrument (Thermo, USA, #PIKOREAL96). Data were analyzed with 2^-ΔΔCt^ value calculation.

### Cell counting kit-8 (CCK-8) assay

U251 cells and SHG-44 cells were seeded in 96-well plates and cultured in RPMI-1640. Cell proliferation index was measured at 0, 12, 24, 48 and 72 h using Cell Counting Kit-8 (Dojindo, Japan, #CK04-500).

### SA-β-galactosidase (β-gal) staining

β-gal staining was performed using the β-Galactosidase Staining Kit (Beyotime Biotechnology, Shanghai, China, #C0602) according to the manufacturer’s instructions. Briefly, the cell culture medium was removed, and the 12-well plates were washed once with PBS, before the addition of 1 ml -galactoside staining fixative. The cells were fixed at room temperature for 15 minutes followed by the removal of the cell fixation solution, and washing with PBS or HBSS 3 times for 3 minutes each. PBS was then removed and 1 ml of working staining solution added to each well. The 12-well plates were sealed with plastic wrap to prevent evaporation and incubated at 37° C overnight. The number of positive SA-β-gal cells was determined and photographed using a light microscope (Olympus, Japan, #CX41-72C02).

### Plate colony formation assay

SHG-44 cells and U251 cells were seeded in 6-well plates at a density of 200 cells/well and incubated at 37° C and 5%CO_2_ for 2 weeks. Incubation of the cells was stopped when the cell colonies were visible to the naked eye. The cells were stained with Giemsa (Solarbio, Beijing, China) and the number of colonies was computed. The results were representative of 3 independent experiments.

### Cell cycle analysis

After transfection, the cells were washed with PBS, and fixed with ethanol at 4° C overnight. Thereafter, 150 μL PI solution (Sigma, USA, #25535-16-4) was added at 4° C for 30 min in the dark. The distribution of the cells in the different cell cycle phases was analyzed using a flow cytometer (Beckman, USA, #A00-1-1102). PI was activated using 488 nm argon lasers and received using a 630 nm filter.

### Statistical analysis

R software (version 3·5·3) was used to conduct statistical analysis.

The two-tailed Students’ t-test was used to identify the differences between groups, and multiple groups were compared using one-way ANOVA test. Besides, to assess the PH assumption, the Schoenfeld residual plots was performed. In the consensus clustering analysis, we used the partition around medoids algorithm. Furthermore, the chi squared test was conducted to analyze clinical differences between the two clusters. To compare the OS, DSS and PFI of glioma cases (high and low-risk score, cluster 1/2), the Kaplan-Meier survival analyses with log-rank test was performed. To analyze the association between two variables, we used Spearman rank and considered P < 0.05 as statistically significant.

## RESULTS

### The expression profiles of aging-related genes in glioma patients and LASSO cox regression analysis

**Figure 2:**
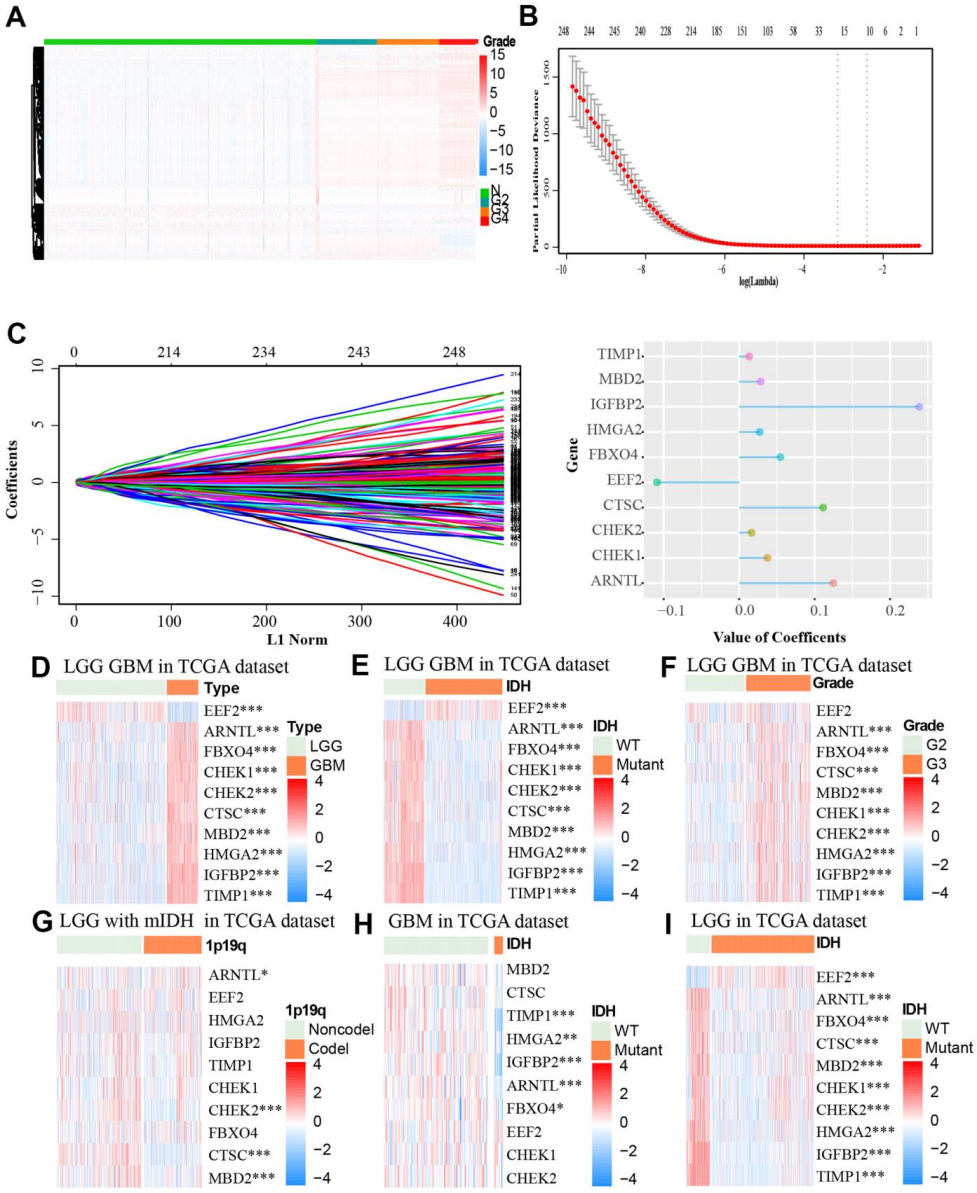
(**A**) The association between the expression level of aging-related genes and grade of tumor based on the TCGA dataset was showed by heat map. (**B**, **C**) LASSO coefficients of the aging-related genes for OS were calculated. The genes with the 10 highest scores are shown. (**D**–**I**) The heat maps, based on the TCGA database, showed downregulated mRNA (blue) or upregulated mRNA (red) of the ten aging-related genes in the subgroups.* p < 0.05, *** p < 0.001.

We retrieved 321 aging-related genes from an aging-related gene dataset from GSEA, out of which, 312 genes were found in TCGA. We then performed univariate cox regression analyses and identified 249 genes that are closely associated with aging (p < 0.05). We retrieved the gene expression profiles of the 249 genes for normal and glioma samples from the TCGA datasets. As shown in Figure 2A, there are distinct differences between the normal samples and glioma samples with different grades. The higher the tumor grade of the samples, the higher the expression of the aging-related genes. After introducing aging-related genes into LASSO Cox regression model, we identified ten most significant aging-related genes and their coefficients including EEF2, ARNTL, FBXO4, CHEK1, CHEK2, CTSC, MBD2, HMGA2, IGFBP2 and TIMP1. The risk score was calculated based on these genes expression condition (Figure 2B, 2C). Out of the 19 genes, CTSC, IGFBP2 and ARNTL had the highest coefficient.

We then retrieved the expression profiles of the 10 genes from glioma samples of different clinical features from the TCGA dataset. The expression levels of ARNTL, FBXO4, CHEK1, CHEK2, CTSC, MBD2, HMGA2, IGFBP2, TIMP1 were higher, while EEF2 expression was lower in GBM tissues compared to LGG (Low-grade gliomas) tissues (Figure 2D). Similar differences were identified between wildtype IDH group and mutant IDH group of the TCGA LGG GBM (the LGG and GBM cases form TCGA) cohort and TCGA LGG cohort. Furthermore, these differences in expression profiles were also observed between groups of different WHO grades except for the EEF2 gene (Figure 2F). There were no obvious differences in expression levels of these genes between mutant IDH group and wildtype IDH group in the GBM cohort (Figure 2D–2I). Moreover CHEK2, CTSC, MBD2 were down-regulated in LGG with 1p19q non-codeletion and mutant IDH (Figure 2G).

### Construction, characteristics and functions of risk score model

**Figure 3:**
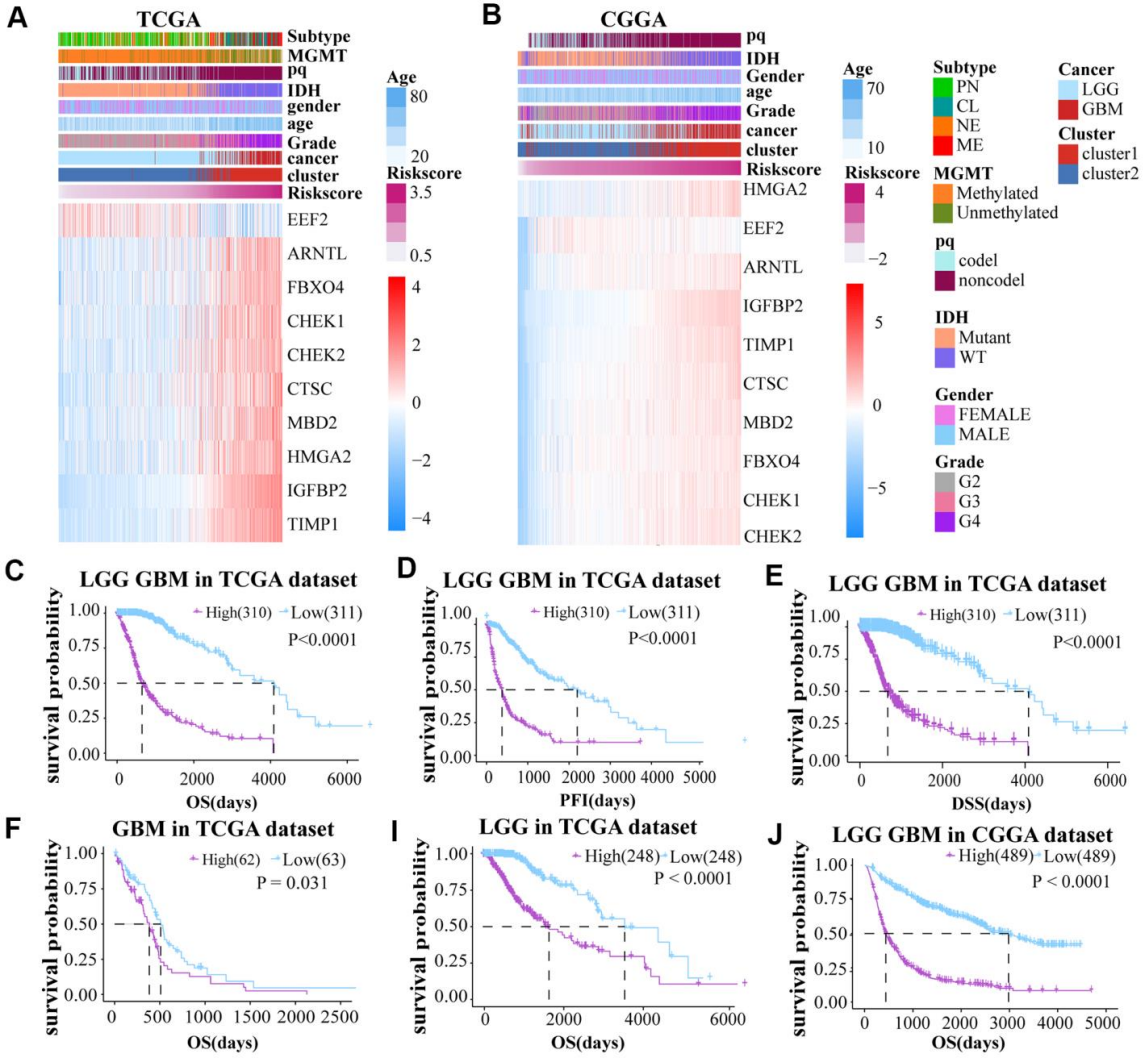
(**A**, **B**) The risk score model was established on basis of aging-related genes. (**C**–**E**) Prognosis (OS, PFI and DSS) of high and low risk score patients (LGG and GBM together) based on TCGA. (**F**–**J**) The OS of GBM and LGG glioma cases from TCGA dataset and LGG GBM glioma cases from CGGA dataset.

A risk score model was constructed on basis of LASSO Cox regression analysis. The expression profiles of aging-related genes were displayed using heat maps for both the TCGA and CGGA datasets. A Volcano Plot of the two groups (high and low-risk) was generated (Supplementary Figure 1F). The samples were divided into two groups according to the median risk score to further investigate the prognosis (Figure 3A, 3B).

To explore the prognostic differences between the two groups, we compared the OS, PFI, and DSS between these groups. Low-risk score cases had obviously longer OS, DSS, and PFI than cases with high risk score in the TCGA LGGGBM, GBM, LGG cohorts (Figure 3C–3I and Supplementary Figure 3C–3F). The high risk score was also associated with shorter OS in the CGGA datasets (Figure 3J and Supplementary Figure 3G–3H). In conclusion, cases with high risk score have worse prognosis.

**Figure 4:**
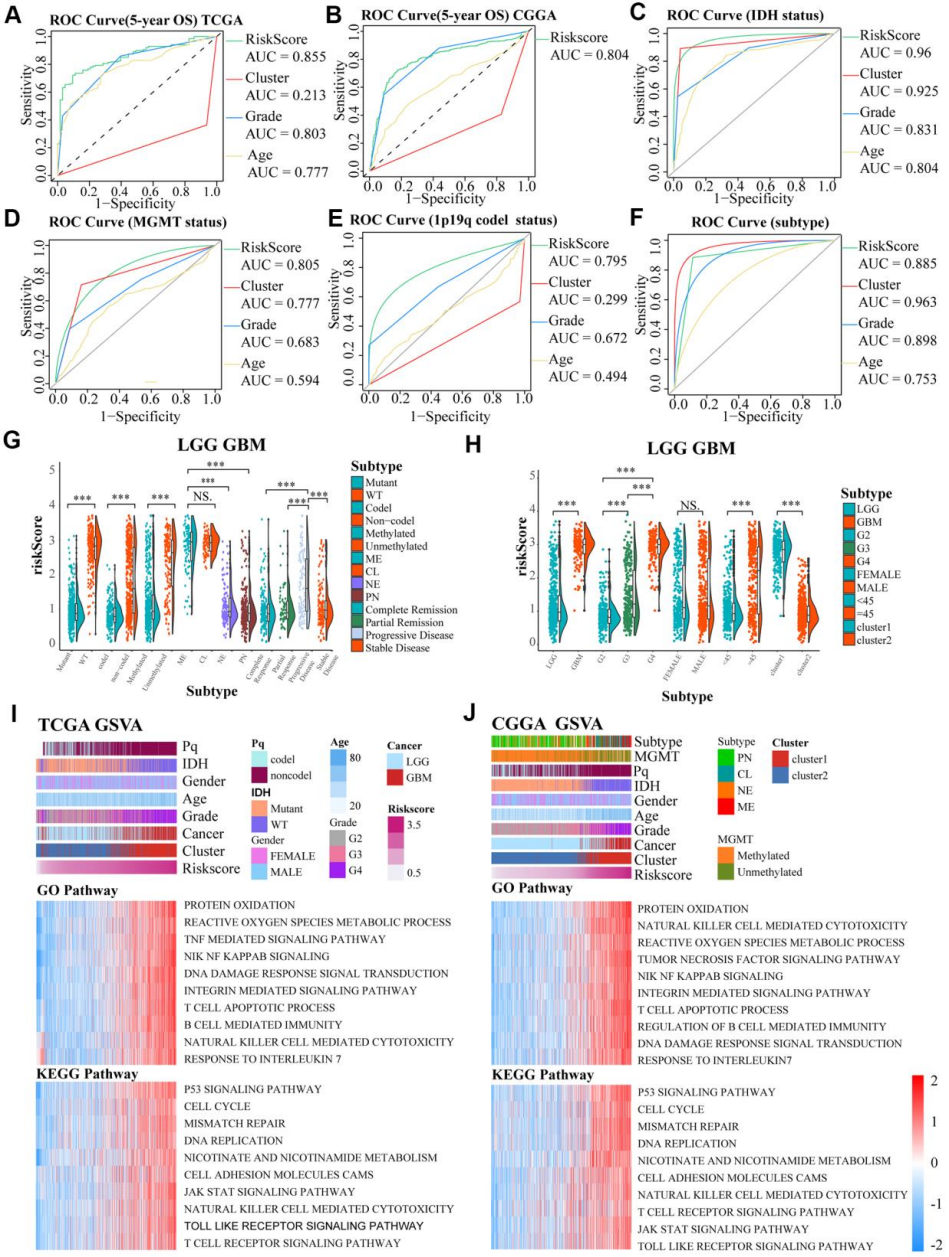
(**A**–**F**) ROC curves exhibited the predictive effect of the four indicators on the clinical characteristics including 5-year OS, IDH status, MGMT status, 1p19q codel status and subtype of glioma cases. (**G**, **H**) The differences in risk scores between subgroups classified by IDH wildtype, 1p19q noncodel, MGMT promoter unmethylated, subtype, GBM groups, higher grades, age, and cluster of LGG and GBM patients based on the TCGA dataset. (**I**, **J**) GO and KEGG analyses for the risk scores using GSVA. The gene set enrichment of several pathways (lower two panels), and distribution of clinical features, clusters, and risk scores (upper panel) were exhibited by the heat map based on TCGA and CGGA. NS. p > 0.05, *** p < 0.001.

To further ensure the predictive effect of the risk score model, the OS of the low-risk and the high-risk groups were also compared by TCGA GBM chip. In addition, survival analysis was also performed in several glioma data sets, including GSE4271, GSE4412, GSE13041, GSE16011, GSE43289, GSE43378, GSE61335, GSE68838, GSE74187, GSE83300, GSE108474 (Supplementary Figure 3I–3Q). The results of these analysis showed that the survival of cases with low risk-score are better than the survival of high risk-score cases. ROC analysis was used to compare the roles of cluster, risk scores, grades, and age in predicting clinical features and prognosis on basis of CGGA and TCGA datasets. We found that risk score was the best factor in predicting 5-year OS, 5-year PFI, 5-year DSS and the clinicopathological features of the gliomas (Figure 4A–4F). These findings indicated that the model was a good predictor of the prognosis of glioma patients.

In TCGA LGGGBM datasets, we found that a high risk score was associated with IDH wildtype, 1p19q noncodel, unmethylated MGMT promoter, subtype, progressive disease, GBM groups, higher grades, age≥45, and cluster1. However, there was no obvious differences between mesenchymal and classical subgroups and between groups separated by gender (Figure 4G, 4H). Moreover, we also observed these significant differences except for the MGMT promoter status between the mesenchymal and classical subtypes in the TCGA LGG (LGG cases from TCGA) cohort, but not in the TCGA GBM (GBM cases from TCGA) (Supplementary Figure 3A, 3B). In general, risk score was closely related to clinical features of the tumors.

The potential functions of the aging-related genes in gliomas were determined using GSVA analysis on basis of TCGA and CGGA datasets (Figure 4I, 4J). Gene set enrichment scores of the pathways were positively correlated with the risk scores, and 10 signaling pathways having high correlation coefficient and statistical significance were selected respectively in GO and KEGG pathways, such as T cell apoptotic, protein oxidation, susceptibility to natural killer cell mediated cytotoxicity, tumor necrosis factor mediated signaling pathway, DNA damage response signal transduction by p53 class mediator, B cell mediated immunity, integrin mediated signaling pathway, response to interleukin 7, reactive oxygen species metabolic process, integrin mediated signaling pathway, response to interleukin 7, NIK/NF kappab signaling, reactive oxygen species metabolic process, p53 signaling pathway, mismatch repair, nicotinate and nicotinamide metabolism, DNA replication, T cell receptor signaling pathway, cell cycle, JAK/STAT signaling pathway and Toll like receptor signaling pathway.

### Genetic mutation and risk score

**Figure 5:**
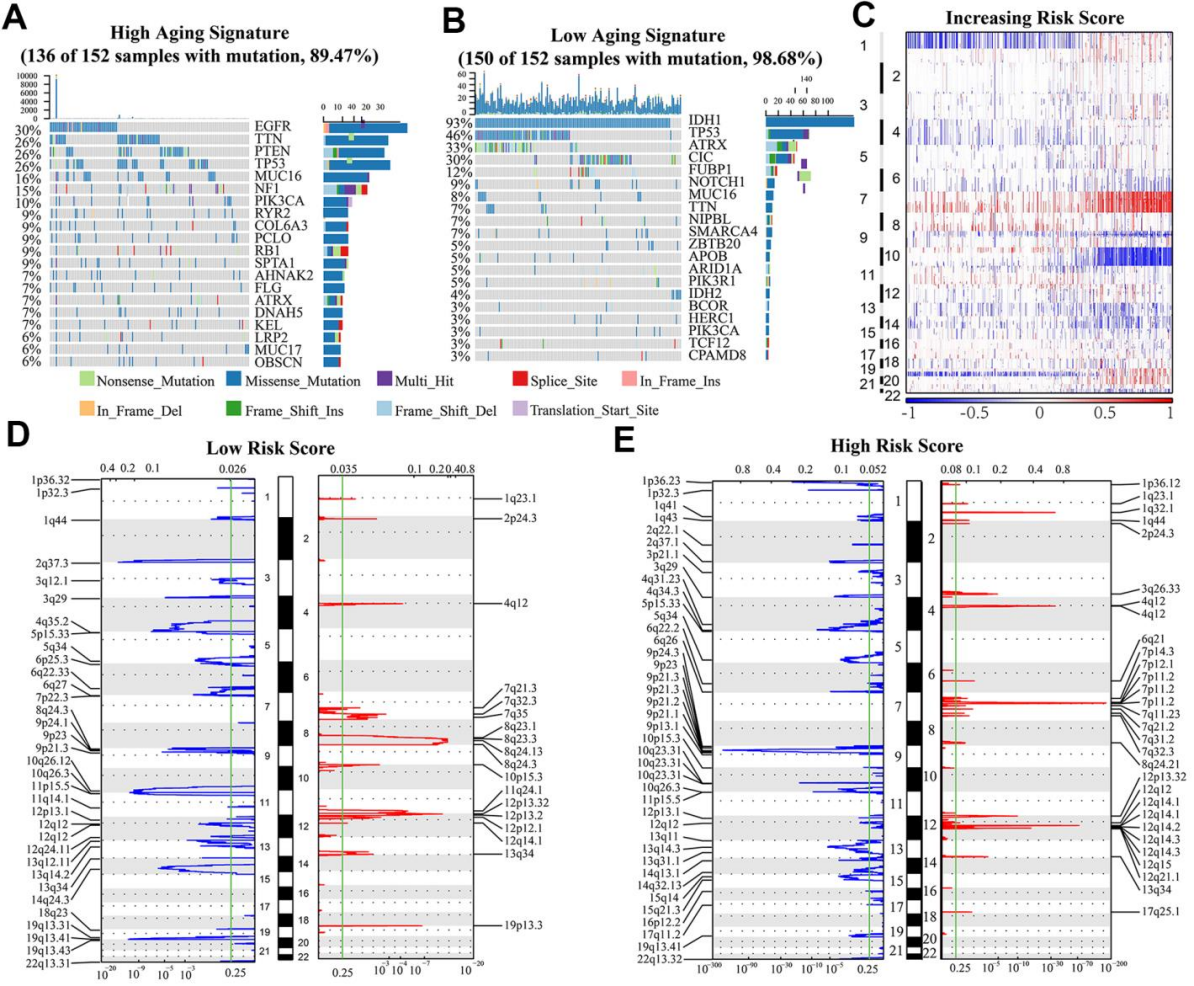
(**A**, **B**) Genes with the highest mutation frequency in high and low risk groups. The overall CNAs profiles (**C**) were shown according to risk score. (**D**, **E**) Chromosomal regions that were significantly amplified (red) and deleted (blue) were identified using GISTIC 2.0 analysis. The threshold for significance was represented by the green line (q value=0.25).

To further investigate the effect of aging-related genes on gliomas, we analyzed the genetic mutations of these cases. We observed somatic mutations in 136 (89.5%) and 150 (98.7%) of the top 20% high risk score cases (152 cases) and top 25% low risk score cases (152 cases), respectively. Some genes showed mutations in both groups (high and low-risk score): TTN, ATRX, TP53, MUC16, and PIK3CA. The frequency of mutations for PIK3CA, MUC16 and TTN was significantly higher in high risk glioma cases (TTN, 26% vs. 7%; MUC16, 16% vs. 8%; PIK3CA, 10% vs. 3%). The mutation frequency of ATRX and TP53 was lower in high risk glioma cases (TP53, 26% vs 46%; ATRX, 7% vs 33%). We also identified mutations in EGFR (30%), PTEN (26%) and NF1 (15%) in high risk group, and IDH1 (93%), CIC (30%) and FUBP1 (12%) in low risk group (Figure 5A, 5B).

SCNAs were compared between the low and high-risk samples to investigate the role of genetic alterations in oncogenesis. As the risk scores increased, the incidence of Chr 7 amplification and Chr 10 deletion increased, while incidence of 1p/19q codeletion reduced (Figure 5C). GISTIC 2.0 analysis also showed that many regions harboring multiple oncogenes such as 12q14.1(CDK4), 7p11.2(EGFR), 4q12 (PDGFRA), and 1q23.1 (PIK3C2B) were amplified in the high risk group. Focal deletion peaks including 1p36.23 (TNFRSF9, ERRFI1), 1p32.3 (CDKN2C), 10q23.31 (PTEN, KLLN), and 9p21.3 (CDKN2A) were also discovered in high-risk group. The genes found in the regions with focal deletions can inhibit the occurrence and development of cancer. On the contrary, there were no significant focal deletion and amplification peaks in the low risk group, and the G values of them were dramatically lower in these cases (Figure 5D, 5E). Besides, there were significant regions of deletion (1p36.23, 2q37.1, 4q34.3, 6q26, 10q23.31, 13q14.3) and amplification (1q32.1, 3q26.33, 7p11.2, 12q15) detected only in the high-risk subgroup (Figure 5D, 5E).

### Construction of cluster model using consensus cluster analysis

To explore the prognostic value of the aging-related genes, we used consensus clustering analysis to divide the tumor samples from the two datasets into two groups (Cluster1 and Cluster2) (Supplementary Figure 4E–4L). Cluster 1 was associated with 1p19q noncodel, unmethylated MGMT promoter, higher grade, IDH wildtype, and GBM and higher risk score (Supplementary Figure 1A, 1B). Principal component analysis (PCA) showed the differences in expression of aging-related genes between cluster 1 and cluster 2 and the results were displayed using a Volcano Plot (Supplementary Figure 1E). There were several genes such as IGFBP2 that showed statistically significant differences in expression between clusters.

There were significant differences in prognosis between 2 clusters in addition to the differences in clinical features and genes expression. Cluster 2 was obviously associated with longer OS, DSS and PFI compared to cluster 1 for LGG and LGM of the TCGA and CGGA (Supplementary Figure 2A–2L).

Sankey diagrams showed that high risk score glioma patients mainly enriched in the wildtype IDH group and cluster 1 and had higher tumor grade, while the low-risk score was correlated with mutant IDH group, cluster2 and lower tumor grade (Supplementary Figure 4A, 4B).

### Immune cells infiltration and risk score

**Figure 6:**
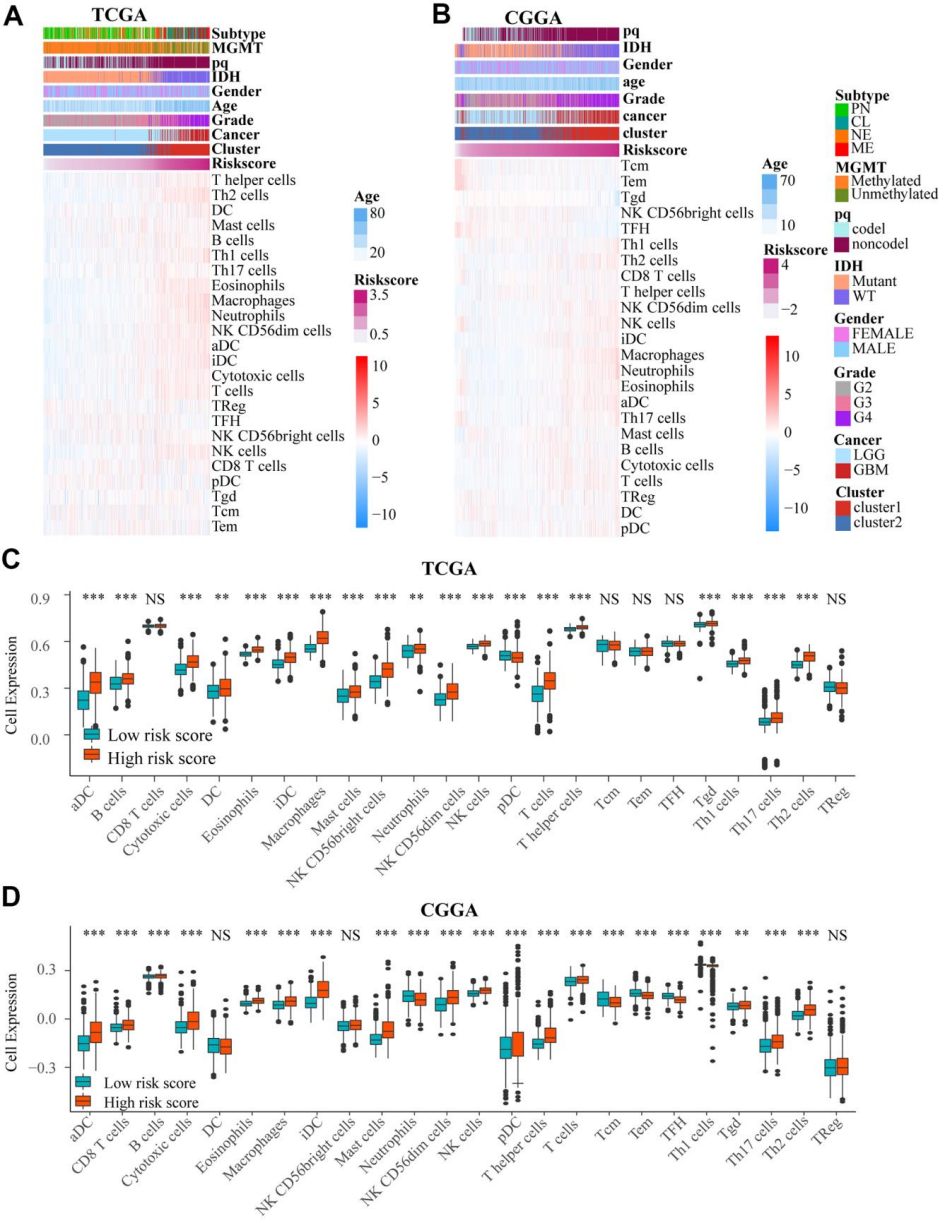
(**A**, **B**) Heat maps show the amount of immune cells and clinical features by ssGSEA based on data in TCGA and CGGA. (**C**, **D**) There was an obvious difference in immune cells number between low and high risk groups in TCGA and CGGA. NS. p > 0.05, ** p < 0.01, *** p < 0.001.

We used heat maps to show the number of immune cells in samples from TCGA and CGGA datasets. We found that the number of a variety of immune cells was associated with risk score (Figure 6A, 6B). We then investigated the difference between low and high risk group by single sample gene set enrichment analysis (ssGSEA) based on TCGA and CGGA. And there was a significant difference in the number of immune cells between the two groups (Figure 6C, 6D and Supplementary Figure 5A, 5B), including aDC, B cells, Th1 cells, DC, cytotoxic cells, mast cells, NK CD56+ cells, T help cells, Tgd, Th17 cells, Th2 cells pDC, NK cells, and macrophages. Among these immune cells, macrophages had the biggest association with risk score (correlation = 0.73) in TCGA and CGGA. In addition, there was also an obvious difference in number of immune cells between 2 clusters in the two datasets (Supplementary Figures 3A, 3B, 5C, 5D).

### Glioma patients in the high-risk group may be more responsive to immunotherapy

**Figure 7:**
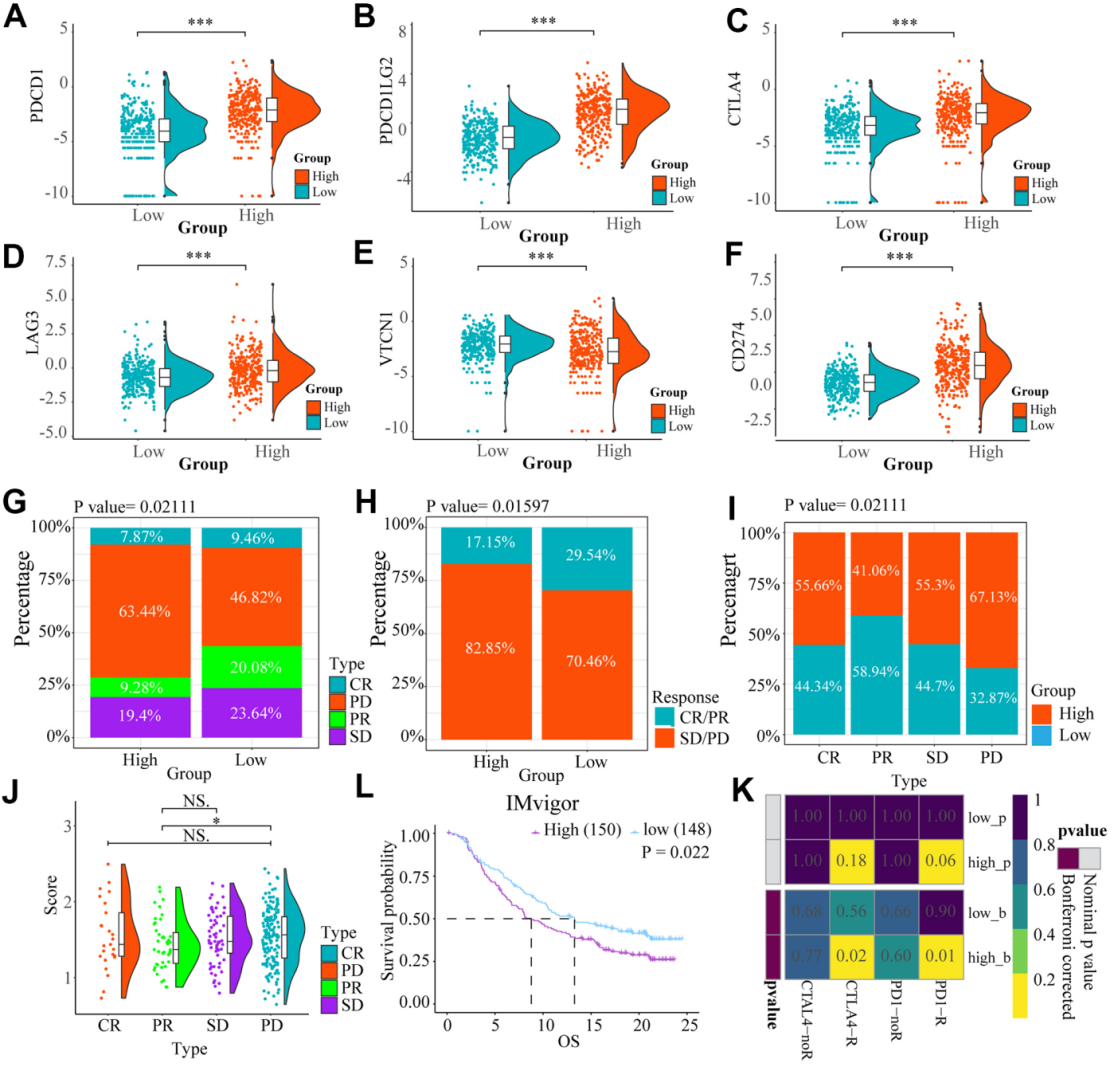
The heat maps (**A**–**F**) showed the different expression conditions of immune checkpoints in low and high-risk groups. (**G**, **H**) The bar charts showed the distribution of the prognosis of cases in high and low-risk score group based on IMvigor datasets. (**I**, **J**) These pictures showed the risk score distribution of cases with different prognosis in IMvigor datasets, (**L**) The OS of cases in high and low risk score groups from IMvigor dataset. (**K**) Submap analysis indicated that patients with high risk score could be more responsive to anti-CTLA-4 (Bonferroni corrected P = 0.02) and anti-PD-1 therapy (Bonferroni corrected P = 0.01) based on TCGA datasets. NS. p > 0.05, * p < 0.05, *** p < 0.001.

We compared expression levels of immune checkpoints between high and low risk groups by heat maps (Figure 7A–7F). Although VTCN1 had lower expression levels in the high risk group (P<0.001), We identified several immune checkpoints including PDCD1LG2, CD274, LGA3, CTLA4 that had higher expression levels in the high risk group (P<0.001).

We explored the effect of the differences in expression of ICBs on the response to immunotherapy between the low and high risk groups by Submap analysis based on TCGA. We found that the cases in the high-risk group had potential to be more responsive to anti-CTLA-4 (Bonferroni corrected P = 0.02) and anti-PD-1 therapy (Bonferroni corrected P = 0.01) compared to low-risk score patients (Figure 7K).

However, based on IMvigor datasets, Progressive disease (PD) portion was larger in high-risk group, while the partial response (PR) patients were more in the low-risk group. However, there is no important difference in stable disease (SD) and complete response (CR) parts between the two groups (Figure 7G, 7H). For patients with different prognosis, the high-risk score part is bigger in PD group than PR group (P<0.05) (Figure 7I, 7J). A comparison of the prognosis of the two groups after treatment with immune checkpoint inhibitors, showed that OS of low-risk score group was higher than high-risk score (Figure 7L) (P = 0.022).

### CTSC inhibition aggravates cell senescence

**Figure 8:**
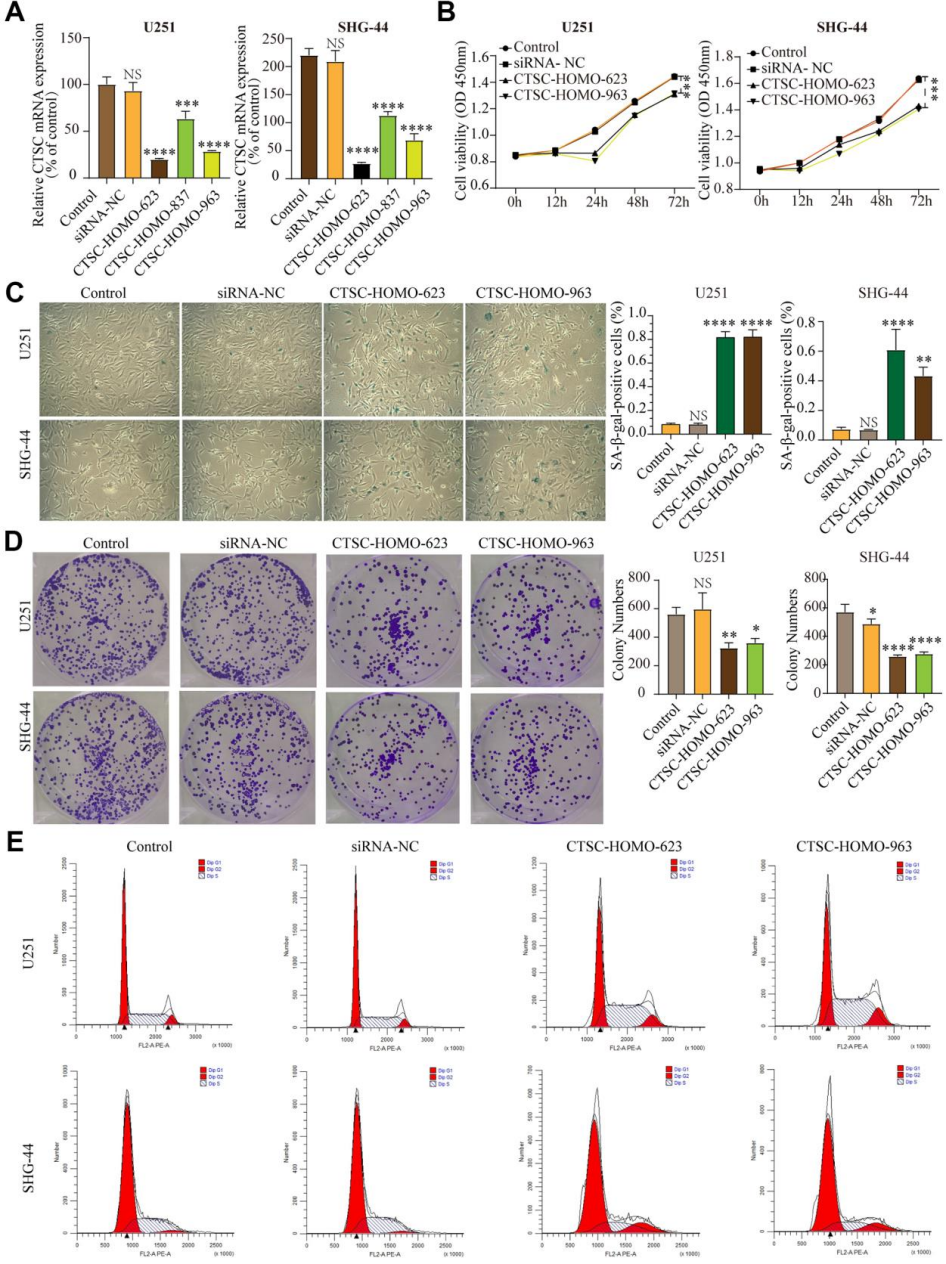
(**A**) The expression levels of CTSC mRNA decreased after siRNA transfection in SHG-44 and U251 cells. (**B**) CCK-8 assays showed that inhibition of CTSC suppressed proliferation of SHG-44 and U251 cells. (**C**) Numbers and images of positive SA-b-gal staining cells in control, siRNA-NC, CTSC-inhibition cells are shown. (**D**) Images and histograms showing colony formation and numbers in the SHG-44 and U251 cells. (**E**) Diagrams showing the percentage distribution of SHG-44 and U251 cells stained with PI in the different phases of the cell cycle. (siRNA-NC, siRNA negative control, NS, p>0.05, * p < 0.05, **p<0.01, ***p<0.001, ****p<0.0001).

According to LASSO Cox regression model, CTSC is one of the most meaningful aging-related genes with highest coefficients. Although previous studies indicated that CTSC expression is up-regulated in several tumor cells, such as pancreatic cancer, hepatocellular carcinoma and breast cancer, its relationship between aging and gliomas is unclear. To study the effect of the CTSC gene on cell senescence in glioma cells, we knocked-down the CTSC gene in SHG-44 cells and U251 cells using siRNA-623, siRNA-837 and siRNA-963. RT-qPCR was used to test the expression levels of CTSC mRNA (Figure 8A) after transfection with the siRNAs. All the transfected cells showed a decrease in CTSC expression (P<0.001), with the cells transfected with siRNA-623 and siRNA-963 showing more significant decrease in expression than those transfected with siRNA-837. We then conducted CCK-8 (Figure 8B) and β-galactosidase staining (Figure 8C) experiments. Inhibition of CTSC for 12h, 24h, 48h and 72h significantly decreased the proliferation of both cells compared to the control group. In addition, CTSC inhibition increased β-gal staining but decreased colony formation in both U251 and SHG-44 cells (Figure 8D). We used flow cytometry to determine the effect of CTSC inhibition on cell cycle progression. We found that the inhibition of CTSC affected the percentage of cells in the G1, G2 and S phases (Figure 8E), which may further influence the progression of cells into senescence. Taken together, these results indicate that CTSC inhibition may lead to cell senescence.

To explore the effect of CTSC expression on the survival of glioma patients, we compared the OS, PFI, and DSS of cases from TCGA and CGGA datasets based on the expression of CTSC. We discovered that the cases with low CTSC expression had obviously longer OS, DSS, and PFI than cases with high CTSC expression in the TCGA LGGGBM, LGG, GBM cohorts except the OS of GBM cases in TCGA (Supplementary Figure 6A–6I). Similarly, high CTSC expression was also associated with shorter OS in the CGGA datasets (Supplementary Figure 6J–6L). These findings indicated that high expression of CTSC might affect prognosis of glioma patients.

## DISCUSSION

Incidence of CNS (central nervous system) tumors has been increasing at a rate of about 1%-2% every year during the last 30 years, especially among the elderly population [1, 32]. The mean overall survival (OS) of GBM patients is approximated to be only 15 months [1, 4], and their five-year survival rate is only 6.8% [1, 6]. Age is one of the most significant predictive factors of glioma occurrence and prognosis for all types and grades of gliomas [24, 25]. Therefore, there is need for further research into this disease. Many aging-related genes are associated with tumors. A study by Dunlap et al. implicated IGFBP2 in the progression of glioma by activating PI3K/Akt pathway [33]. In addition, CHEK2*1100delC heterozygosity is related to increased risk for several neoplasms such as breast cancer [33]. However, there have been no studies that have explored the effects of the aging-related genes on the clinical outcome and progression of gliomas.

In our present study, we constructed aging risk score and cluster models of glioma based on aging-related genes using bioinformatics analysis, LASSO Cox regression analysis and consensus clustering analysis. The efficacy of cluster and risk score in predicting the clinical features and prognosis was investigated and compared. The predictions of prognosis of the two models were consistent with the facts. Furthermore, we discovered obvious differences in expression of several genes between two groups, which is consistent with previous reports [34].

Somatic alterations analysis showed that high risk score was associated with mutations of oncogenes (PIK3CA, MUC16, TTN), but had less mutations of ATRX and TP53. Furthermore, amplification peaks of oncogenes (PIK3C2B, PDGFRA, EGFR, CDK4), and deletion peaks of tumor suppressor genes (TUSC1, CDKN2A, CDKN2B, PTEN, FAS, BNIP3) were detected in the gliomas with a high risk score. These findings revealed that the PDI family are involved in the malignant biological process in gliomas. GISTIC 2.0 analysis revealed that many regions harboring oncogenes were amplified in the high risk group. The genes found in the regions with focal deletions can inhibit the occurrence and development of cancer in high-risk group. However, there was no significant focal deletion and amplification peaks in the low-risk group, and the G values of them were dramatically lower. These indicated that expression of aging-related genes might lead to the mutations of a lot of genes related to malignant biological process.

To investigate the mechanism of action of aging-related genes in gliomas, we conducted GSVA analysis, and identified common biological functions of aging-related genes in development and tumorigenesis of gliomas, including P53 signaling pathway, DNA damage response, natural killer cell mediated cytotoxicity, tumor necrosis factor (TNF)-mediated signaling pathway, which are consist with previous studies [35, 36]. The enrichment of the Arf/p53 pathways potentially had a significant influence not only on the accumulation of cellular damage and aging, but also on the surveillance and suppression of tumors [15]. Hui-Ling Ou et al. reported that DNA damage not only drives the aging process but also causes cancer development [37].

We also discovered several signaling pathways associated with the aging-related genes in gliomas that had not been previously reported. These pathways included NF-κB signaling, Cell cycle, Apoptosis, toll-like receptor signaling pathway, and JAK-STAT pathway. Previous studies had identified chronic increase in inflammatory signals as a hallmark of aging and as a significant activator of NF-κB target genes [38]. NF-κB mediated inflammation had been studied and regarded as a biomarker of aging [38, 39]. Carmela Rita Balistreri reported the functional importance of toll-like receptor4 signaling pathway in evoking aorta aging and disease [40]. Furthermore, previous studies showed that cell cycle is involved in brain aging process [41].

Another significant finding was that aging was related to immune regulation in gliomas, including regulation of T cell apoptotic process, T cell receptor signaling pathway, natural killer cell mediated cytotoxicity, and B cell mediated immunity. Previous studies have shown that immune injury is a common hall-mark of cancer and aging [13]. Immunosenescence is known as changes in adaptive and innate immune systems during aging [42]. Many cells (such as Th1 responses, CD8 cytotoxic T cells, B cells, NK cells, and macrophages) are involved in Cancer immunosurveillance which is the ability of immune system to identify and kill new malignant cells [43, 44]. However, malignant cells may gradually gain the ability to evade the immune system with the aging progress, which may lead to tumor progression [42].

In this study, we found that the degree of aging was related to the infiltration of various immune cells such as Th cell, Tcm cell, DC, CD8+ T cell, which indicates the promising role of these genes in immunotherapy. These findings showed that the immune response and the immune system could be studied more to understand the function of aging in gliomas, and the application of aging-related genes in cancer therapy.

The relationship between immune cells and risk score was also investigated. Several immune cells such as macrophages, dendritic cells, and T cells were closely associated with the risk score. Both macrophages and dendritic cells play important roles in the tumor microenvironment [45], while the T cells play an important role in immunotherapy [46]. The results from this study indicated that aging-related genes may affect the tumor microenvironment by regulating immune cells and stromal cells, thus contributing to the development of tumors.

We further explored the prognostic effect of risk score model for glioma patients treated with immunotherapy. We found that patients in high-risk group had higher expression of immune checkpoints and were more responsive to anti-PD-1 and anti-CTLA-4 therapies than the patients in low-risk group. However, our another analysis indicated that patients with bladder urothelial carcinoma having low risk score had longer OS and better prognosis than patients in high risk group based on IMvigor dataset. These indicate that the glioma patients in high-risk group might respond better to immunotherapy, but it need to be further investigated.

Cathepsin C (CTSC) is a lysosomal cysteine protease [47] and a member of aging-related genes. CTSC expression is up-regulated in a variety of tumor cells, such as 0. Several studies have shown that the down regulation of CTSC can inhibit the development of tumors [27, 48–50]. This study found that the inhibition of CTSC increases cell senescence and the expression of CTSC is associated with poor prognosis of glioma patients. We therefore postulated that CTSC promotes tumorigenesis and progression of tumors by reducing cell senescence. However, the relationship among CTSC, gliomas and aging is not clear. We found that inhibition of CTSC increases cell senescence, and previous studies have reported that high state of senescence inhibits the development of cancer. Therefore, CTSC might promote tumor development by inhibiting cell senescence. Previous studies have shown that CTSC plays an important role in the regulation of autophagy, which may also be the mechanism of promoting tumor death [27]. However further studies are required to determine the relationship between CTSC and autophagy.

There have been reports on the inhibitors of aging by some studies. Herein, we briefly review some of these inhibitors. Wu et al. found that metformin may fight cancer and aging by restricting transit of RagC GTPase through the nuclear pore complex [51, 52]. Rapamycin is able to inhibit aging by inhibiting mTOR but has some negative side effects [53, 54]. Julie Chao et al. revealed that kallistatin regulates aging and cancer by affecting the expression levels of miR-34a and miR-21 [55].

In summary, this study constructed clinical models concerning aging-related genes in clinicopathological characteristics and predicting prognosis of glioma patients and discovered potential signaling pathways. In the current study, we analyzed the expression profiles, prognostic value, and potential mechanisms of action of aging-related genes in gliomas. However, further research should be conducted on the relationship between aging and cancer, and on the validation of biological function of aging-related genes in gliomas.

### Data availability statement

Data availability Public data for tumor cases is available in TCGA, CGGA, GEO and IMvigor.

## Supplementary Material

Supplementary Figures
