# Supplementary material for: Aging-related genes are potential prognostic biomarkers for patients with gliomas
Source: Aging (Albany NY). 2021 May 4;13(9):13239–63. doi: 10.18632/aging.203008 (PMC8148480; doi:10.18632/aging.203008)
Supplement: Supplementary Figures [file aging-13-203008-s001.pdf]

SUPPLEMENTARY FIGURES

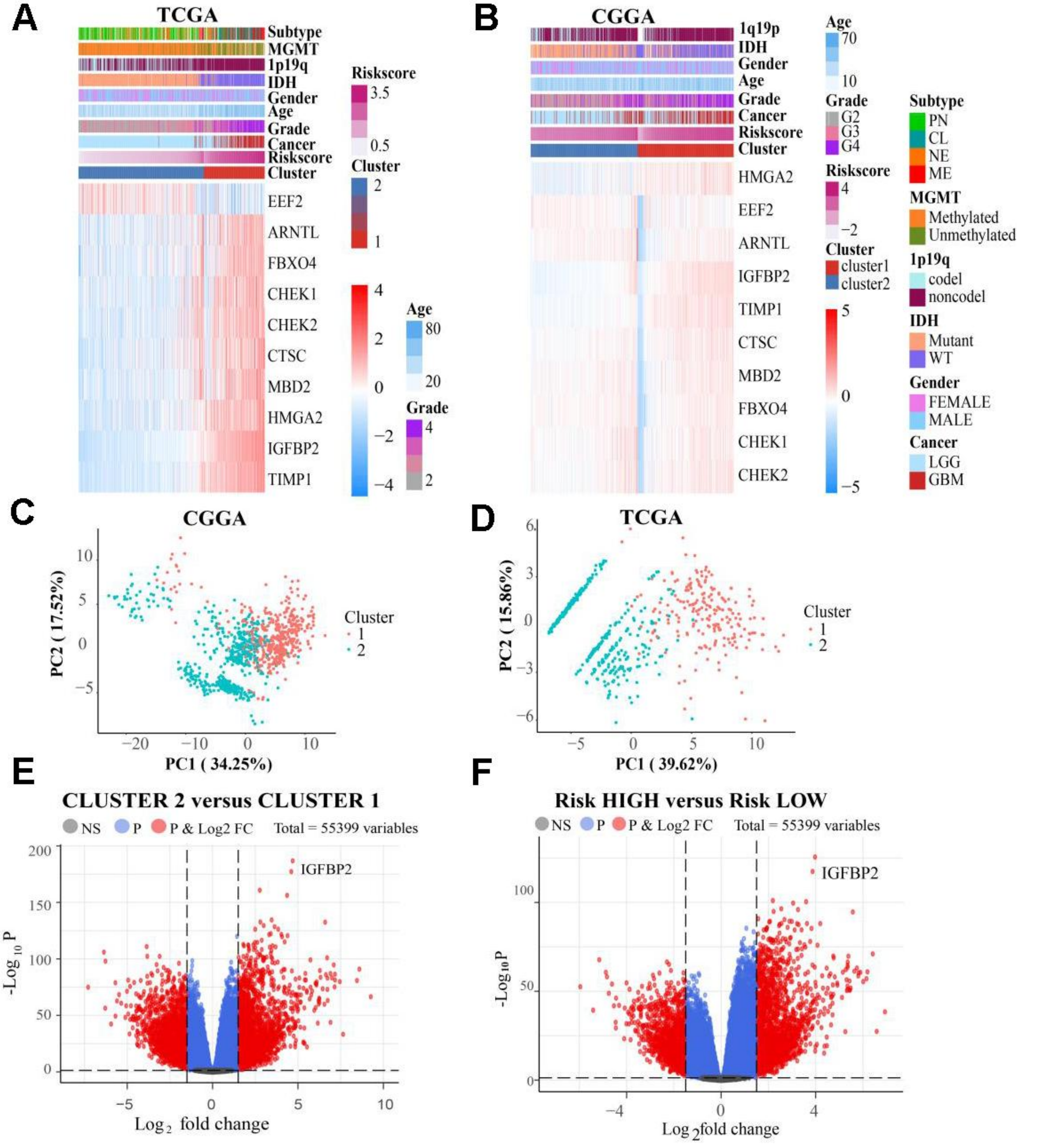

Supplementary Figure 1. (**A**, **B**) The expression levels of aging-related genes and clinical features between cluster 1 and cluster 2 were displayed by heatmaps. Principal component analysis (PCA) revealed the difference of aging-related genes mRNA expression between two clusters in TCGA (**C**) and CGGA (**D**). (**E**, **F**) The Volcano Plot of the two clusters and risk-high and low groups exhibited the different genes expression between them.

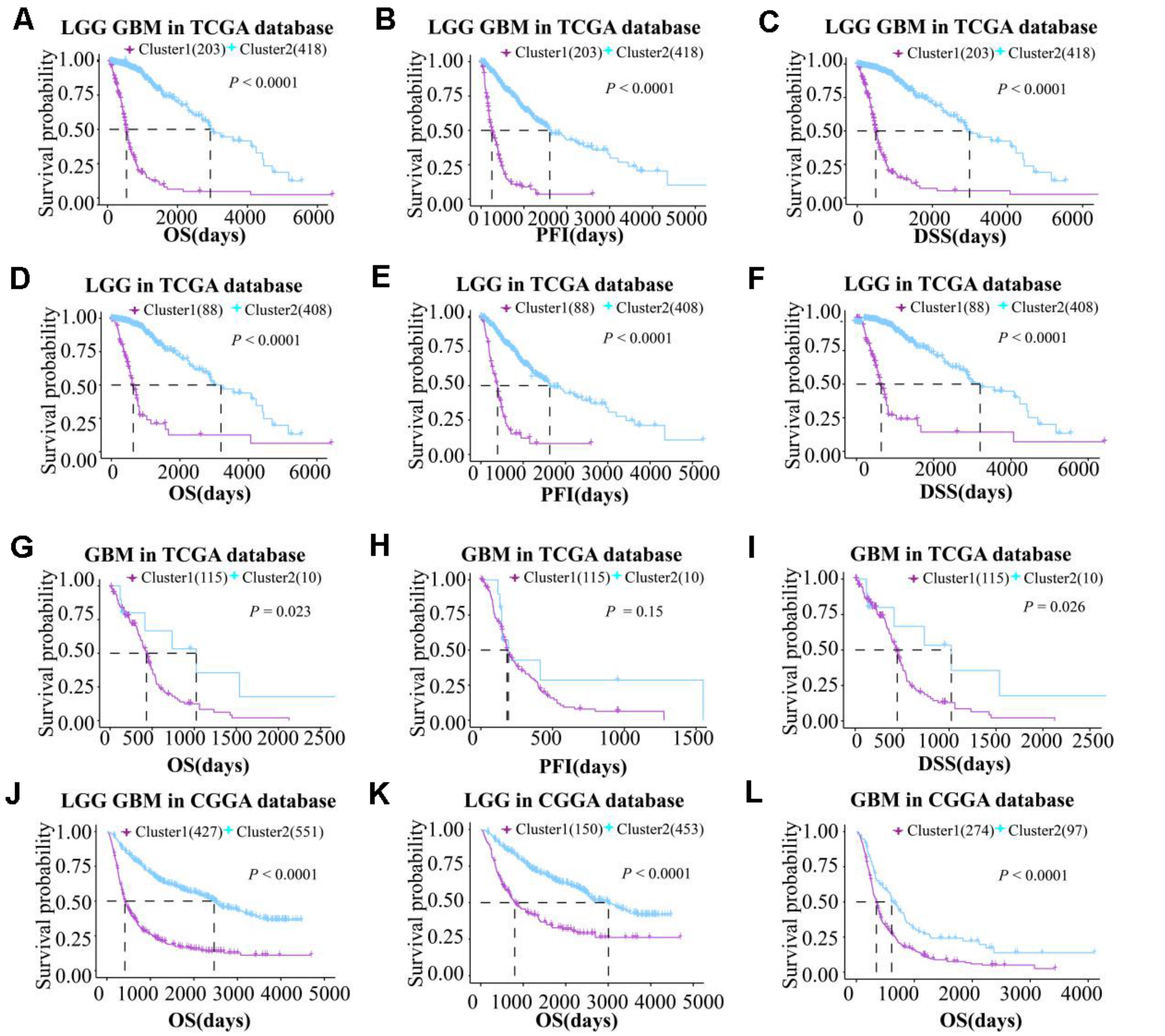

Supplementary Figure 2. The prognostic value of aging-related genes in gliomas patients. The survival curves were displayed based on clusters for the OS, PFI and DSS in LGGGBM (**A**–**C**), LGG (**D**–**F**), and GBM (**G**–**I**) patients from TCGA. (**J**–**L**) Kaplan-Meier survival curves of OS were constructed based on the CCGA datasets.

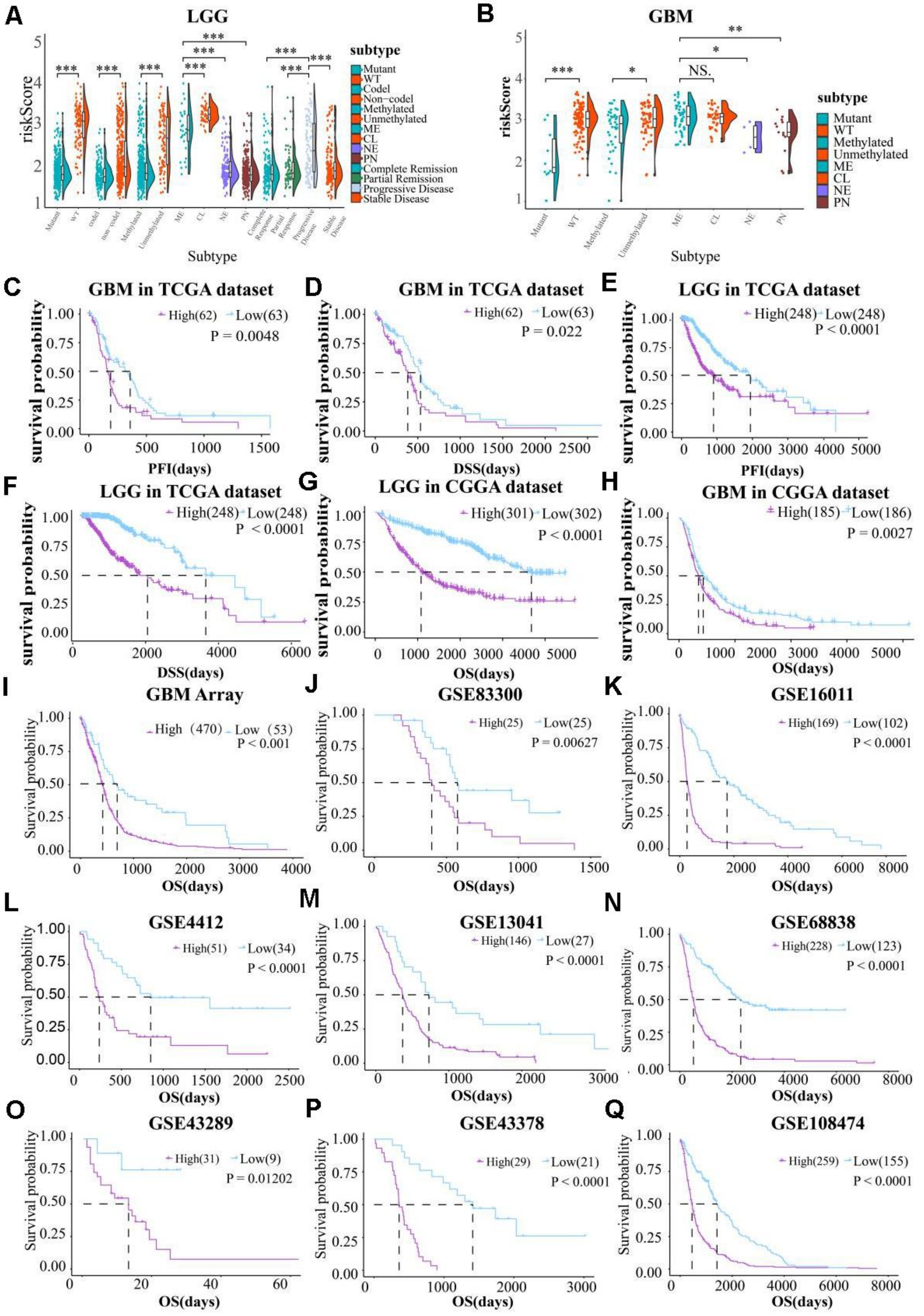

Supplementary Figure 3. (**A**, **B**) The risk scores differences between subgroups classified by IDH wildtype, 1p19q noncodel, MGMT promoter unmethylated, subtype, GBM groups, higher grades, age, and cluster of LGG and GBM patients according to the information from TCGA. The prognostic value of risk scores in gliomas patients. Kaplan-Meier survival curves for the PFI and DSS in GBM (**C**, **D**) and LGG (**E**, **F**) samples. (**G**–**Q**) The survival curves of OS were constructed based on the CCGA datasets and nine datasets from GEO. NS. $p > 0.05$, *** $p < 0.001$.

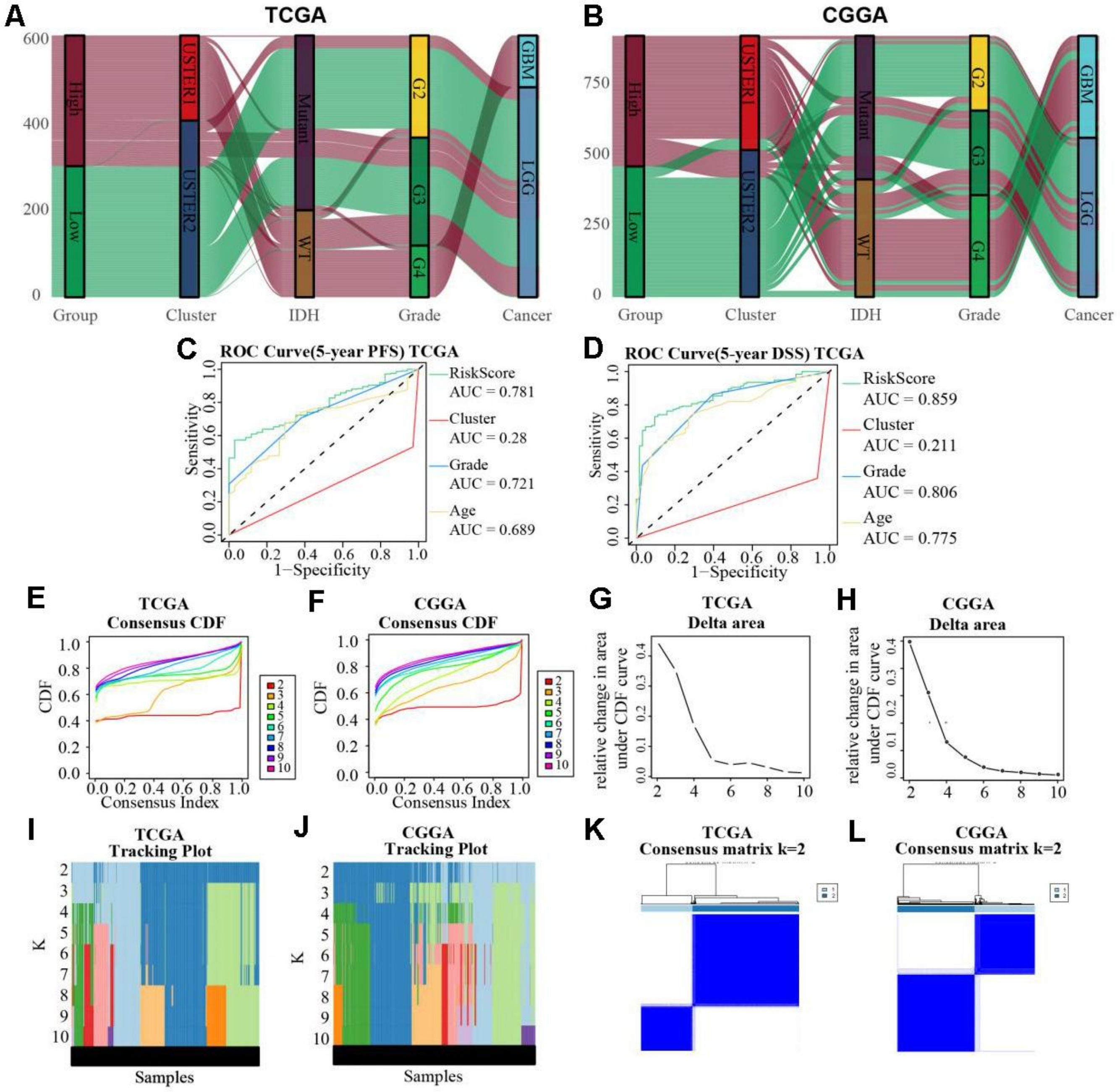

Supplementary Figure 4. (**A**, **B**) The relationship among the five indicators, cluster, cancer type, grade, risk score, and age, using TCGA and CGGA. The predictive role of risk score, cluster, grade and age was compared in the PF, DSS, subtype, MGMT promoter status, IDH status, and 1p19q codel status of glioma patients based on TCGA and OS based on CGGA as the validation set. (**C**, **D**) ROC curves showed the predictive effect of the four indicators on the clinical characteristics including 5-year PFS and DSS of gliomas cases. (**E**–**L**) Gliomas cases from TCGA and CGGA were clustered by the consensus clustering algorithm. The cumulative distribution function (CDF) plot of the aging-related genes mRNA expression in gliomas from TCGA and CGGA were showed. k=2 was defined as the optimal number.

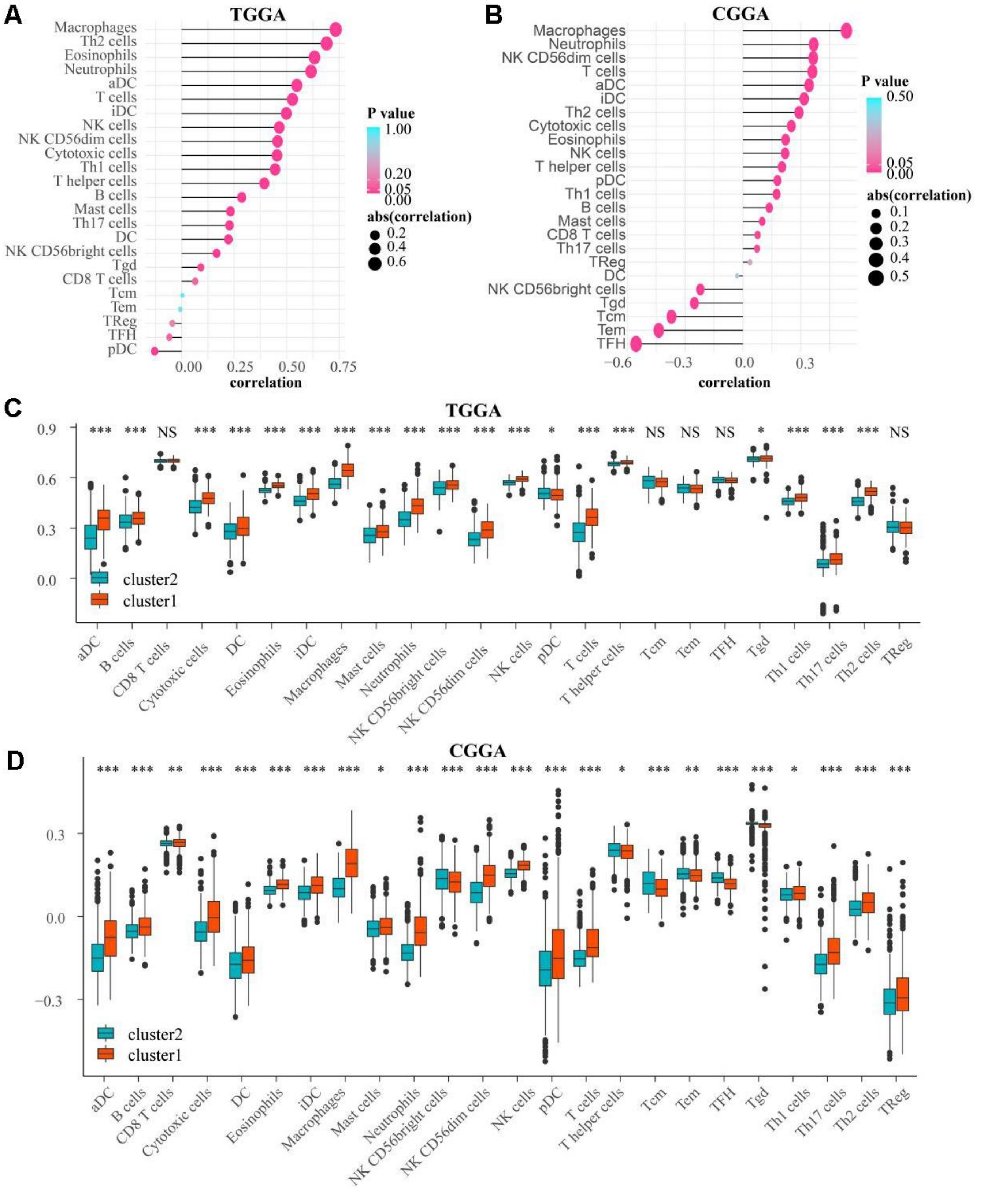

Supplementary Figure 5. (**A**, **B**) The correlation between immune cells and risk score. (**C**, **D**) There were also differences in the number of immune cells between cluster1 and cluster2 both in TCGA and CGGA. NS. p > 0.05, * p < 0.05, ** p < 0.01, *** p < 0.001.

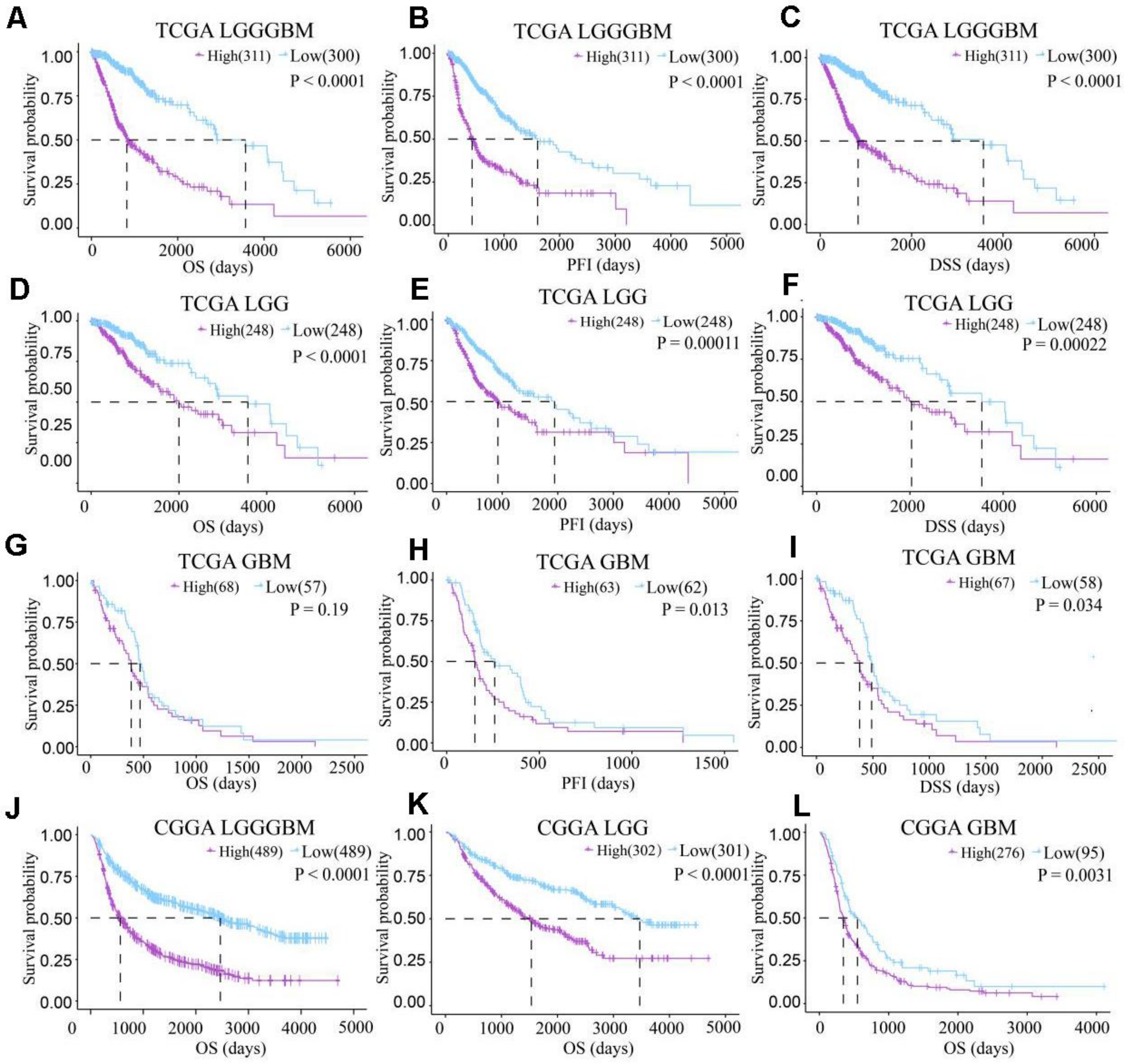

Supplementary Figure 6. The differences of prognosis between CTSC high expression and CTSC low expression cases. Kaplan-Meier survival curves of high and low CTSC expression cases were displayed for the OS, PFI and DSS in LGGGBM (**A**–**C**), LGG (**D**–**F**), and GBM (**G**–**I**) patients from TCGA. (**J**–**L**) The survival curves of OS were also constructed based on the CCGA datasets.
